# Experimental Assessment of OSNMA-Enabled GNSS Positioning in Interference-Affected RF Environments

**DOI:** 10.3390/s25030729

**Published:** 2025-01-25

**Authors:** Alexandru Rusu-Casandra, Elena Simona Lohan

**Affiliations:** 1 Telecommunications Department, Politehnica National University of Science and Technology, 060042 Bucharest, Romania; 2 Tampere Wireless Research Center, Electrical Engineering Unit, Tampere University, 33720 Tampere, Finland; elena-simona.lohan@tuni.fi

**Keywords:** Galileo, open service navigation message authentication (OSNMA), message, RF interference, jamming, spoofing, authentication

## Abstract

This article investigates the performance of the Galileo Open Service Navigation Message Authentication (OSNMA) system in real-life environments prone to RF interference (RFI), jamming, and/or spoofing attacks. Considering the existing data that indicate a relatively high number of RFI- and spoofing-related incidents reported in Eastern Europe, this study details a data-collection campaign along various roads through urban, suburban, and rural settings, mostly in three border counties in East and South-East of Romania, and presents the results based on the data analysis. The key performance indicators are determined from the perspective of an end user relying only on Galileo OSNMA authenticated signals. The Galileo OSNMA signals were captured using one of the few commercially available GNSS receivers that can perform this OSNMA authentication algorithm incorporating the satellite signals. This work includes a presentation of the receiver’s operation and of the authentication results obtained during test runs that experienced an unusually high number of RFI-related incidents, followed by a detailed analysis of instances when such RFI impaired or fully prevented obtaining an authenticated position, velocity, and time (PVT) solution. The results indicate that Galileo OSNMA demonstrates significant robustness against interference in real-life RF-degraded environments, dealing with both accidental and intentional interference.

## 1. Introduction

As the number of modern devices and applications relying on GNSS signals continues to grow, so does their susceptibility to radio frequency interference—whether accidental or deliberate. GNSS systems are increasingly exposed to malicious over-the-air attacks, such as spoofing and meaconing, which can manipulate or disrupt GNSS signal reception and compromise the integrity of navigation and positioning data. In order to detect and mitigate these threats, various methods have been developed and implemented, such as signal quality monitoring, receiver autonomous integrity monitoring, multi-constellation and multi-frequency techniques, multi-antenna based systems for direction-of-arrival monitoring and null steering, time-based verification for receiver clock consistency and correlation peak analysis, cross-referencing with external sensors, cryptography-based secure authentication, etc. [1,2]. Initial proposals and proof of concept [3,4] investigated the design and development of a service that could provide navigation message authentication (NMA) for the Galileo GNSS open service signal using cryptography, in a way fully backward-compatible with the specifications and receiver requirements in the existing Signal-In-Space Interface Control Document (SIS ICD) and with minimum changes to the deployed infrastructure. The evolution of this proposed system led in November 2020 to the start of broadcasting test signals of a new service intended to provide to Galileo open service users a reliable proof of the authenticity of the received navigation messages and the identity of the originating satellite vehicle (SV) [5]. This innovative Open Service Navigation Message Authentication (OSNMA) system, developed by the European Union Agency for the Space Programme (EUSPA), represents the first such feature available for civilian use of GNSS signals and has been designed to enable receivers to calculate a PVT fix relying only on Galileo navigation messaging (I/NAV) transmitted over the E1-B signal-in-space (SIS) component and on strong cryptographic mechanisms, without needing access to a trusted third party. The OSNMA capability of the Galileo constellation provides a significant security layer to protect against attacks using falsified or recorded and re-broadcast GNSS signals, such as spoofing and meaconing, contributing to the security and resilience of the vast array of applications that nowadays use satellite navigation.

In November 2021, the operation of the OSNMA system entered the public observation test phase, as the second stage of development and improvement before reaching full service provision in a few years [6]. During this second test phase, EUSPA transmitted a test SIS, disseminating all the necessary cryptographic material and full documentation to allow receiver manufacturers, application developers, and members of the academic community to receive real OSNMA frames from the Galileo space segment for the first time. During the public observation test phase, key stakeholders and interested parties developed software and hardware implementations of the OSNMA algorithm in accordance with the interface control documents (ICDs) and receiver requirements [7,8,9,10] and also published the results of various functional tests, both theoretical and experimental. During this phase, EUSPA also started including a regular measurement of OSNMA key performance indicators relevant at this phase, in the quarterly published Galileo Open Service Quarterly Performance Report [11]. Even if the parameters characterizing the performances of provided OSNMA Service are not yet described by any minimum performance level (MPL), the performance indicators analyzed and presented in the report confirm that the service is operating successfully. On 2 September 2024, EUSPA announced that it had completed the testing of the Galileo OSNMA, declared the conclusion of the public observation phase, and announced the forthcoming declaration of its operational launch [12].

In the almost three years of operation in the test phase, various scientific studies described operational fields tests of OSNMA-enabled GNSS positioning and reported on its overall performance [13,14,15,16,17,18,19]. Some of these are reviewed below.

Initial published reports dealt with the implementation of the protocol, evaluating particular implementations of OSNMA, in terms of both adherence to the algorithm and receiver behavior. They also defined the key performance indicators to evaluate the operation of particular implementation under various tests. One of the earliest such reports [15] described criteria developed at the European Commission Joint Research Center (JRC) facilities in Ispra that were applied to carry out receiver assessment, both in nominal and during special cases, and included a description of the test bench used. The authors also defined and described key performance indicators (KPIs): OSNMA PVT accuracy, PVT availability, time to first authenticated fix (TTFAF), and computational load.

Other authors [16] have shown how different data-retrieval strategies can be implemented by an OSNMA-enabled receiver in order to acquire the elements necessary to authenticate the Galileo I/NAV message. Two main approaches that a receiver is expected to use are described in different combinations. The first design option is the retrieval of data sequentially from one satellite or in parallel from all satellites in view; the second is the retrieval of the OSNMA data on a sub-frame basis or on a page basis. It is concluded that the reception conditions in which the receiver operates, as well as the needs of the targeted application in terms of time to authentication and time between authentications, should be considered when selecting the data retrieval logic.

Another published study [17] presented observed operational information relating to Galileo OSNMA and KPIs from the investigation of a four-day long dataset captured in fixed-position and open-sky scenarios in southern Finland. The research used the proprietary OSNMA implementation developed at the Finnish Geospatial Research Institute, Espoo, Finland, allowing monitoring of dropped navigation pages and unsuccessful cyclic redundancy checks (CRCs). Overall, the analysis concluded that 99.74% of the time, a receiver would be able to determine authenticated fixes, and it also reported KPIs such as the number of simultaneously authenticated satellites over time, percentage of authenticated fixes, and time to first authenticated solution. The paper also reported how satellite visibility and the elevation mask affected those figures.

Other authors [18] focused their study on the accessibility of a high-performance and authenticated positioning system as an essential requirement for the viability of new applications in the transportation domain, including autonomous cars. Their collected measurements included real-time kinematic (RTK) positioning data from open-sky and urban conditions in southern Finland along an approx. 400 km trajectory, using commercial GNSS receivers set to two different Galileo OSNMA authentication modes. The results showed a compromise between accuracy and availability vs. authenticity associated with using GPS and authenticated Galileo satellites vs. only Galileo OSNMA authenticated satellites. The results demonstrated that aiming at a strict authenticated PVT solution resulted in a important decrease in general performance and availability compared with not employing authentication at all, as shown by an important increase in the 95% horizontal error percentiles, in both open-sky and urban scenarios.

The present paper investigates a novel aspect that has been only marginally or not at all addressed in most previous studies, i.e., the performance of the Galileo OSNMA system in real-life environments prone to RF interference, jamming, and spoofing attacks. Our analysis focuses on how road users might utilize a commercial GNSS receiver with the OSNMA algorithm enabled, relying exclusively on authenticated Galileo-based positioning for navigation. The main contributions of this paper are: an overview of the OSNMA protocol as currently supported by few commercial GNSS receivers; conducting an extensive measurement campaign in three counties in Romania close to the eastern border; creating a dataset of logged signal files that will also be made available to other researchers; providing an analysis of jamming and spoofing detection in real-life scenarios on captured signals affected by RFI; and discussion of future paths towards signal authentication and improving GNSS receivers.

This paper is organized as follows. Section 2 provides a general description of the OSNMA protocol, limited to key aspects of interest for interpreting the results of tests. Section 3 presents considerations for selecting the routes of test drives in the data-collection campaign and the equipment setup. Section 4 describes the test scenarios executed and the results obtained after analyzing the collected GNSS signals files. This is followed by discussion of the results in Section 5. This paper closes with the conclusions and ideas for further improvement, discussed in Section 6.

## 2. Brief Description of OSNMA Protocol

The Galileo OSNMA system enables a receiver to authenticate individual satellite vehicles (SVs) by integrating unique features into the I/NAV message broadcast on the E1-B signal, based on strong cryptographic techniques that are resistant to prediction or manipulation by malicious entities. E1-B signals are transmitted at a carrier frequency of 1575.42 MHz. Receivers equipped for authentication apply the corresponding decoding cryptography to these features to differentiate between genuine and counterfeit signals and provide the internal positioning engine with the confirmation that the received Galileo open service navigation message was indeed generated by that constellation. Therefore, OSNMA is a data authentication process and can be considered as the first level of authenticating a position, velocity, and time (PVT) solution [20]. This level should be complemented by authentication at the range level, which verifies the authenticity of the measured distances to the satellites. This level is currently under development as the Commercial Authentication Service (CAS) and is based on the encryption of the E6-C component at signal level to deliver Spreading Code Authentication (SCA) [21]. The Galileo E6-C signal is transmitted at a carrier frequency of 1278.75 MHz. However, at data level authentication, OSNMA cryptography already increases the unpredictability of the E1-B signal. Thus, it also protects the ranging measurements and makes it much more challenging for an attacker to spoof them [22]. Even if OSNMA on its own cannot fully confirm the authenticity of the receiver’s position, through this report, an authenticated PVT solution must be considered as one computed using signals received from Galileo SVs that have passed the I/NAV authenticity check.

**Figure 1:**
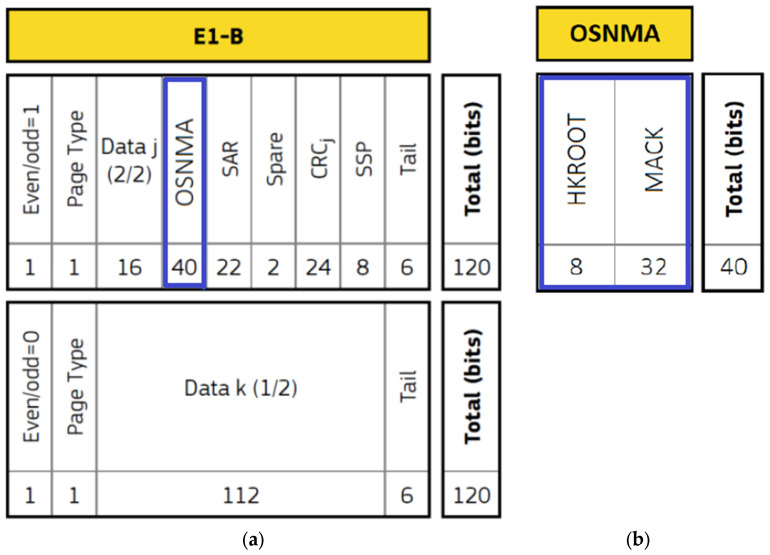
(**a**) I/NAV nominal page with allocation of bits (blue block); (**b**) Structure of the OSNMA data field [7].

The OSNMA data are broadcast inside the Galileo SIS 40-bit OSNMA field, included the E1-B I/NAV navigation message in the odd half page, as described in Figure 1a [7]. It should be noted that, until Galileo constellation started broadcasting OSNMA information, this 40-bit section in each I/NAV page was not in use, being marked as reserved in the SIS ICD.

The OSNMA authentication data are created in the ground segment and transferred to the Galileo satellites. Therefore, only those SVs that are connected to a ground uplink station are broadcasting OSNMA data at any moment. Thus, the OSNMA data are disseminated by only a part of the constellation; the remaining satellites have the OSNMA field filled with a 40-bit sequence of zeroes and the receiver must not use such a field. The group of satellites transmitting the OSNMA data alternates dynamically over time and the user does not know beforehand which SVs are broadcasting OSNMA information. For Galileo SVs that do not transmit OSNMA data, their navigation data can be cross-authenticated by the satellites included in the abovementioned subset [7]. In this way, cross-authentication also increases the redundancy of the system and allows future use of Galileo OSNMA to authenticate satellites from other GNSS space segments, such as GPS.

OSNMA utilizes several thoroughly investigated cryptographic algorithms, adapted to the specific conditions of a low-bandwidth open satellite transmission channel and to the need to minimize the computing power and internal memory requirements imposed on the receiver. The core cryptographic protocol used in OSNMA is a variation of the lightweight TESLA (timed efficient stream loss-tolerant authentication) protocol, which produces a sequence of secret keys through a one-way function such as cryptographic hash algorithms and employs them in reversed order, one key being used to derive the previous key in the chain [7]. This principle is used to authenticate the key against the root key of the TESLA chain. The validity of this root key is in turn checked through asymmetric verification, using a digital signature algorithm and a public key distributed by the system and stored in the receiver. Each TESLA key is afterwards used to produce a shortened message authentication code (MAC), named a tag, which certificates part of the navigation message sent by a satellite in a previous sub-frame.

The 40 bits of OSNMA message are separated into header and root key (HKROOT) (8 bits) and MAC and key (MACK) (32 bits) sections, as presented in Figure 1b [7].

The data accumulate over the course of 15 nominal pages of one I/NAV sub-frame, to form a 120-bit HKROOT and 480-bit MACK messages. A brief explanation of the content of the two messages presented in Figure 1b is as follows [20]:

- The HKROOT message starts with an 8-bit NMA header segment, followed by a 112-bit digital signature message (DSM) section, consisting of a DSM header followed by a DSM block. The DSM can be of two types: the DSM-PKR, providing the public key, or the DSM-KROOT, providing the TESLA chain parameters and the digital signature of the TESLA chain root key. The DSM is transmitted in blocks over several sub-frames and is repeated across different satellites, so that pages from satellites transmitting the same block can be merged to assemble the complete DSM block;
- In each sub-frame and for each satellite, the MACK section is used to transmit, a number of tags with their relative information (Tag and Info field) and the TESLA chain key. All satellites transmitting OSNMA data broadcast the same TESLA chain key in the same sub-frame. Also, all satellites transmit the same key at the same epoch. However, the tags are different, with each tag authenticating a specific part of the navigation message of a specific satellite transmitted at a specific time. Taking into consideration that part of the navigation message is repeated over several sub-frames, it is possible for different tags to authenticate a similar navigation message.

**Figure 2:**
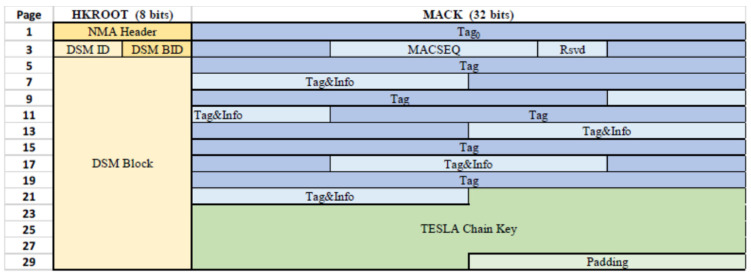
Complete content of HKROOT and MACK sections accumulated after 15 odd half-pages of one sub-frame in I/NAV, for the OSNMA public test configuration [20].

Figure 2 represents the content of both the HKROOT and MACK sections over one full I/NAV sub-frame, taking as an example the OSNMA configuration broadcast for the public testing phase.

**Figure 3:**
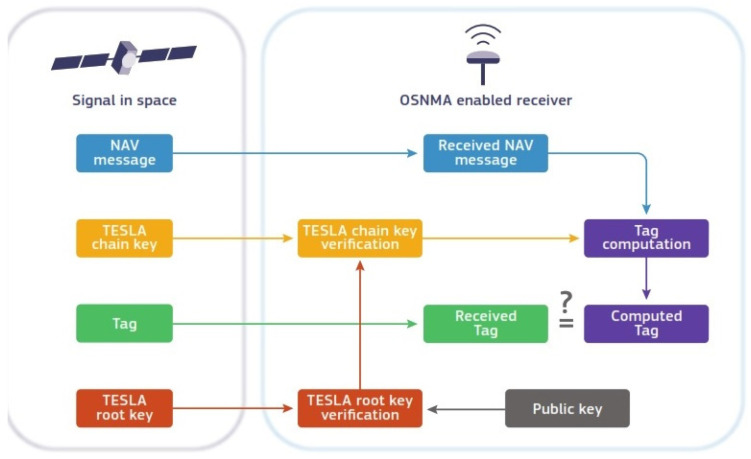
Data processing and authentication decision logic in OSNMA [9].

An OSNMA-compatible receiver has to perform the main steps of data processing described in Figure 3 [9]:

- First, the receiver collects both the navigation data and the associated OSNMA elements, which include the tag, the TESLA chain key, and the TESLA root key. The tag authenticates the navigation data that were transmitted before it, and it is authenticated using the corresponding TESLA chain key received later;
- The TESLA root key is validated using its digital signature, which is decrypted through a public key stored in the receiver;
- The receiver then uses the TESLA root key or an already verified chain key to authenticate the TESLA chain key;
- After that, the receiver generates a tag locally, using the verified TESLA chain key and the corresponding data, and compares it with the received tag;
- If all these verifications succeed, the receiver can trust that the navigation data are authentic.

Currently, several GNSS receiver manufacturers, such as Septentrio (Mosaic X5 and Polar X5) and U-Blox, have successfully integrated the necessary functionalities to decode Galileo OSNMA data and can execute the authentication algorithm within their products. This demonstrates growing industry support for this pioneering security enhancement [23,24]. Furthermore, software developers have also implemented OSNMA processing capabilities, offering both proprietary, such as Rokubun (Medeea and Spear tools) [25], and open-source solutions (software receivers from Finnish Geodetic Institute, Frauenhofer Institute, KU Leuven University) [13,26]. This dual approach ensures that users have a variety of tools to authenticate GNSS signals for improved positioning and navigation security.

## 3. Data-Collection Campaign

### 3.1. Selection of Test Routes for Field Measurements

Advance planning of the test drive routes was necessary in order to cover a reasonably large terrain area under conditions similar to those encountered by an ordinary user traveling in a vehicle equipped with a Galileo receiver. To fulfill the aims of this study, our experimental investigation had to include operational tests of OSNMA-enabled receivers in areas where the presence of RFI of various types, produced unintentionally or intentionally, could be encountered. For this purpose, an analysis was carried out of the increasingly numerous reports of RFI incidents reported by the crews of aircraft or maritime vessels operating in the area of the eastern border of Romania and in the Black Sea basin.

The period since the beginning of 2022 has been marked by numerous incidents of intentional RFI (jamming) and even falsification of the GNSS signal (spoofing) observed in the eastern area of Europe and attributed by most analysts to armed conflicts and the use of radio electronic warfare. Our study did not aim to investigate the sources of interference or the categories to which they belong. It focused on identifying some locations or areas where RFI was stronger and to varying degrees affected the reception of GNSS signals by road civilian users. This paper also concentrates on the operation of the OSNMA system.

**Figure 4:**
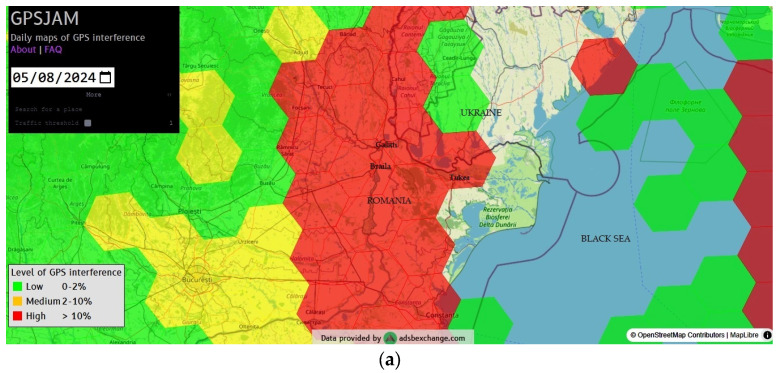

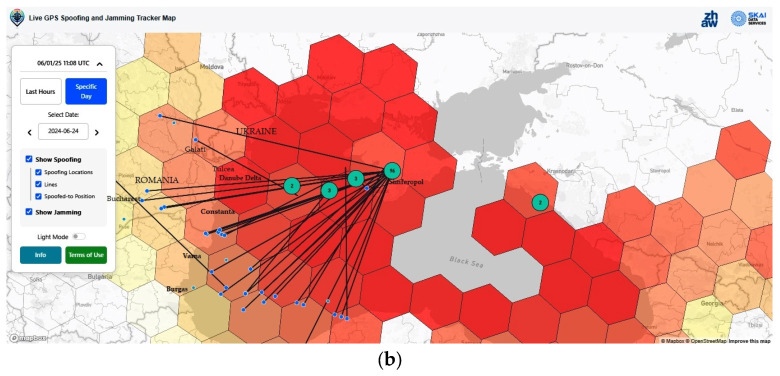
Areas with low navigation accuracy and significant RFI incidents during flights in the Black Sea basin in May–June 2024 (**a**) Red hexagons show where more than 10% of aircraft reported low navigation accuracy [27] (**b**) The cluster indicates areas where spoofed GPS positions of aircraft have been detected. The number (96) within the blue circle shows how many flights were spoofed at that specific location [28].

In general, the loss of GNSS signal can cause downgrading of aircrafts’ positional computation capabilities. Reports of temporary GNSS positioning loss that could be attributed to RFI incidents are aggregated in several databases openly accessible via the internet, which also pinpoint on maps the areas in which they occurred [27,28]. The hexagons in red on the maps in Figure 4 indicate where more than 10% of aircraft declared decreased satellite navigation accuracy in the Black Sea region. These are usually correlated with areas of known and suspected RFI incidents and jamming activity. Live automatic dependent surveillance (ADS)-B messages transmitted by aircraft are collected and utilized by the databases ADS-B Exchange and OpenSky Network [29,30]. They identify and display potentially affected aircraft in real time, as well as where GPS jamming activity has been observed during flights.

**Figure 5:**
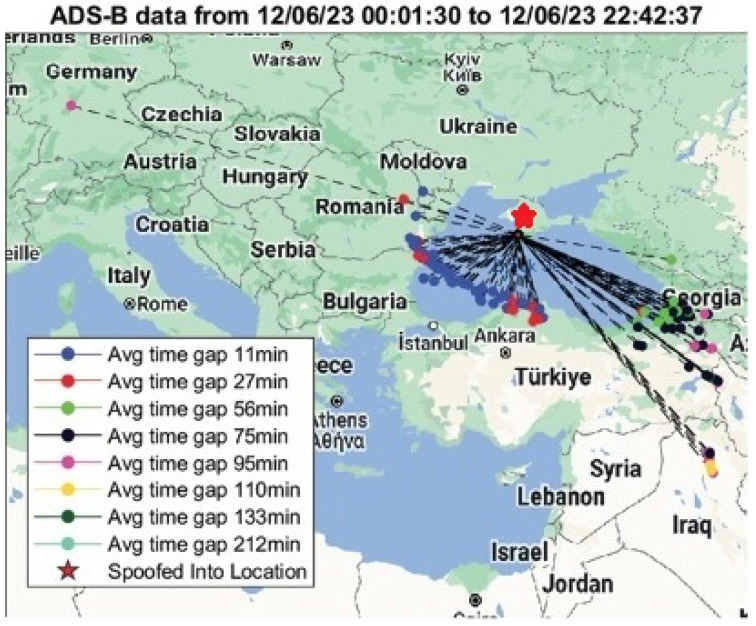
Aircraft locations before, during, and after spoofing on 6 December 2023 [31].

Spoofing of GNSS signals confirmed by ADS-B has been experienced in the last two years in Eastern Europe [31]. A detailed analysis of a spoofing incident over the Black Sea area is presented in Figure 5, showing aircraft positions before, during, and after spoofing on 6 December 2023. The red star in the plot indicates the location where aircraft were spoofed to.

**Figure 6:**
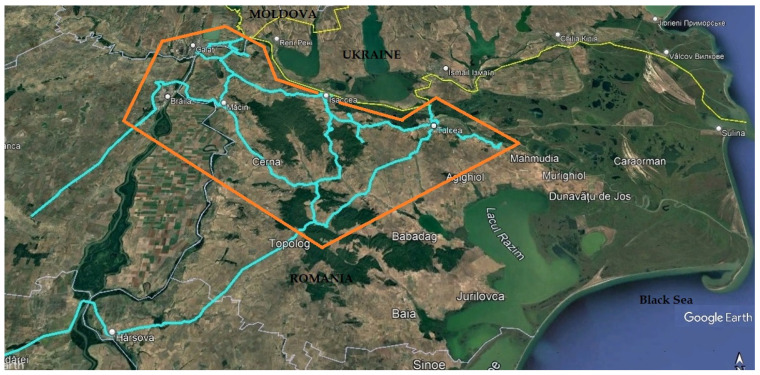
Map of area chosen for data collection campaign in counties Braila, Galati, and Tulcea (orange color polygon) and data-collection test drives actually conducted (cyan).

The data sources and maps presented above (Figure 6) indicate that in Romania, the counties where low navigation accuracy is most probable are Galati, Braila, and Tulcea. These are located on the aircraft corridors where RFI interference or spoofing have been reported. Therefore, the test routes for performing the experimental measurements and collecting the data necessary for our study were planned to travel through the mentioned counties, in the area south of the Ukraine–Romania border, broadly illustrated in Figure 6. It must be noted that the RFIs encountered by an aircraft’s receiver at a particular position do not necessarily also affect the receiver of a user travelling on a road below, due to considerable altitude difference between the two.

### 3.2. Description of Equipment

**Figure 7:**
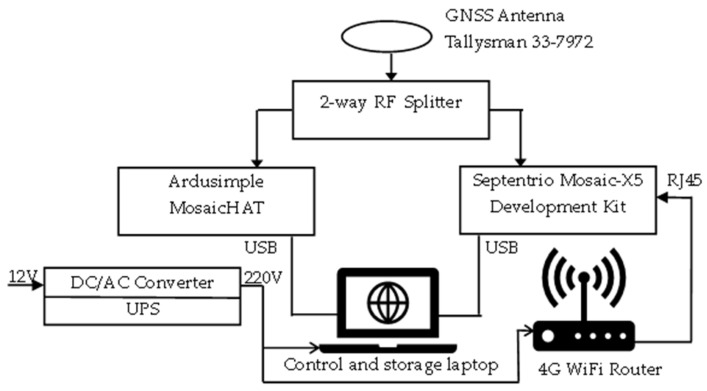
Block diagram of equipment setup used in data-collection campaign.

**Figure 8:**
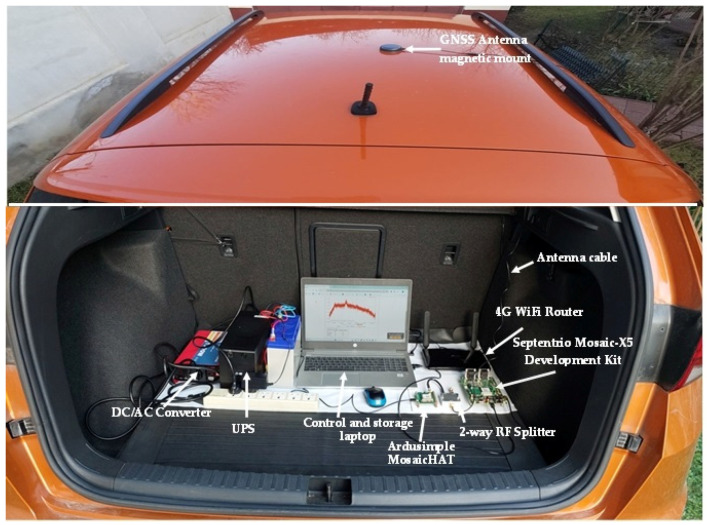
Equipment as installed in trunk of test vehicle. (The image of the rooftop has been superimposed onto the image of the trunk).

The equipment used in the data-collection campaign was installed in a dedicated vehicle according to the set-up described in Figure 7 and Figure 8. A triple-band precision positioning GNSS antenna, model Tallysman 33-7972, was installed on the rooftop of the vehicle. Two GNSS receivers based on the Septentrio Mosaic-X5 module (Leuven, Belgium) were used to receive and process GNSS signals [32,33]. The Mosaic-X5 is one of the few commercially available GNSS receiver modules capable of natively processing the OSNMA protocol. It can operate in three modes for the authentication of Galileo signals, as follows:

(1) OSNMA ‘off’ mode, using all the tracked satellites for calculation of PVT solutions;
(2) OSNMA ‘loose’ mode, using GPS or Galileo satellites that are successfully authenticated or with unknown authentication status, but rejecting satellites that have failed the authentication protocol.
(3) OSNMA ‘strict’ mode, using only successfully authenticated Galileo satellites.

The first receiver, Ardusimple MosaicHAT board [32], was configured to receive Galileo and GPS satellites and operate in OSNMA loose mode. The second receiver, a Septentrio Mosaic-X5 Development Kit [26] was configured to receive only Galileo satellite signals and compute the PVT solution in OSNMA strict mode. The logged data in OSNMA loose mode were not analyzed for this study, as this report focuses exclusively on strictly authenticated Galileo-based positioning.

The elevation mask for both receivers was set to 5 degrees. For optimal OSNMA decoding, it is generally advantageous to have data from as many Galileo SVs as possible available for processing and not discard valuable data blocks from low-elevation satellites [18].

Both receivers were configured to use real-time correction messaging (RTCM)for real-time kinematic (RTK) positioning, retrieved via the networked transport of RTCM via internet protocol (NTRIP), connecting to the Romanian ROMPOS reference stations network. Three reference stations closest to the location of the test vehicle were used throughout the tests. A 4G router provided internet access for NTRIP and also for connection to a network time protocol (NTP) server to synchronize the internal clock of the receiver operating in OSNMA strict mode.

In order to optimize the computation of the PVT solution for conditions specific to in-motion operation, the dedicated dynamic settings of the two receivers were adjusted correctly to assist in compensating for different types of motion of the vehicle.

Power supply for the equipment was provided by a 12 V/220 V inverter, and a separate uninterruptible power supply (UPS) maintained functional continuity even when the vehicle engine was stopped.

### 3.3. Data-Collection Test Drives

Data-collection campaigns were scheduled for months with favorable weather conditions in the areas of interest, avoiding peak tourist season to minimize the impact of heavy traffic and road congestion on travel time. The test drives were conducted in two sessions, during 30 May–1 June and 13–14 September 2024, driving the test vehicle for 23.9 h, i.e., 86,046 epochs at 1 Hz measurement rate, over a length of approx. 755 km of road in the counties of Braila, Galati, and Tulcea, according to previous planning presented in Section 3.1. The routes travelled, mapped in Figure 6, were chosen so that the data collection vehicle could drive through environments of diverse terrain and encounter real situations of intense road traffic, increasing the possibility of experiencing RF interference. Some of the test drives included intervals of static measurement in areas such as parking lots, near road intersections, or at suitable locations chosen along the navigation waterway of the River Danube. The main roads were travelled in both directions on different days and at different times, so that various signal obstructions or RFI sources could be encountered.

Examining the routes on terrain maps from Google Earth, the total length travelled during the data-collection campaign can be divided in the following categories, according to the classification of environments defined in EN16803-1 [34]:

(1) “Flat rural”, or “clear sky”—rural roads in flat countryside with masking angles smaller than 10°, no mountains nor high hills, amounting to approx. 41% of the total length;
(2) “Tree-lined rural”—rural roads with lines of trees with foliage on each side and a significant effect on signal reception due to the foliage, approx. 25% of the total length;
(3) “Mountainous”—roads with sharp curves and high mountains around, generally on one side of a valley, with numerous tunnels and sometimes trees, and masking angles between 10° and 80°. The terrain category was actually ”hilly” and without tunnels, but with dense tree lines on one or both sides of the road, approx. 4% of the total length;
(4) “European peri-urban”—suburban or medium cities’ ring roads with relatively large streets and buildings of small to medium height, and masking angles up to 30°, approx. 26% of the total length;
(5) “European urban”—standard European “old” big cities with relatively narrow streets but sometimes large avenues or ring roads, with buildings from medium height to tall, and masking angles up to 60° generating frequent multipath and non-line-of-sight (NLOS) phenomena, approx. 4% of the total length.

It is important to observe the high total percentage of roads in categories 2 to 5, which amounted to approx. 59% of the total length travelled and resulted in non-line-of-sight propagation (NLOS), signal obstructions, and multipath effects that were quite apparent within the overall results, as shown later in Section 4.

The data-collection campaign was split into 18 test drives, also labeled as tracks, with the corresponding duration and length traveled listed in columns 3 and 4 of Table 1.

## 4. Results

The GNSS signals monitored during data collection using the on-board GNSS receivers were logged in Septentrio binary format (SBF) files. SBF is the proprietary binary output format of Septentrio receivers; it stores all relevant data, such as satellite observations, navigation messages, signal values and status, PVT solutions, receiver status, and other information, in binary blocks that are referred to as SBF blocks [23]. An SBF file was recorded for each road segment traveled (test driven), which was subsequently analyzed using the software SBF Analyzer v24.0 [35] to produce the plots and the statistical values presented later in this article. Our research focused on the measurements obtained by the Septentrio receiver running in OSNMA strict mode, tracking only Galileo SVs. We did not investigate the performances of the receivers running in OSNMA loose or off mode, as these have already been well documented in the literature [17,18,19].

Table 1 presents the values of interest obtained for each file logged in OSNMA strict mode:

- Presence of RFI in signals logged, estimated as percentage of total number of 1 s epochs in each test drive;
- Availability of OSNMA-authenticated PVT solution or loss of this solution, calculated as percentage of total number of 1 s epochs in each test drive. Only OSNMA strict mode recordings were analyzed. In this mode, PVT is not available at all if fewer than four authenticated Galileo SVs are tracked;
- Mean number of Galileo satellites tracked and mean number of OSNMA authenticated satellites in PVT, calculated for each test drive.

The bottom line of the table contains the total number of 1 s epochs as a total of all 18 test drives, the total length of roads travelled, and the average values calculated over the total number of 1 s epochs in the data-collection campaign.

### Data Content of Logged SBF Files

The data files collected during the test drives were inspected using the SBF Analyzer, which provided detailed time plots referring to all relevant parameters of the received GNSS signals, including values and statistics of the measurements provided by the receiver. The presence of RFI was investigated using the information given by some of these plots, as described in the following three examples, which are illustrative of different situations experienced during the test drives.

Case study no. 1: spoofing

**Figure 9:**
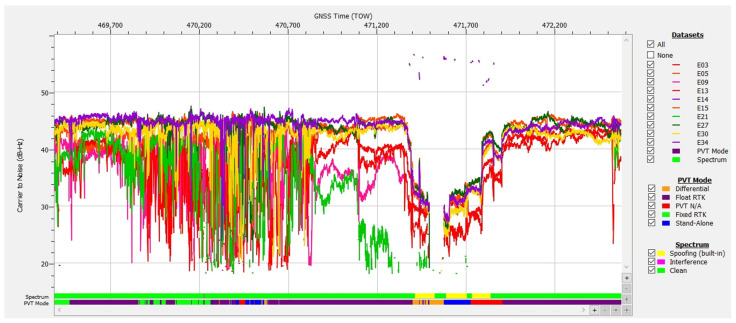

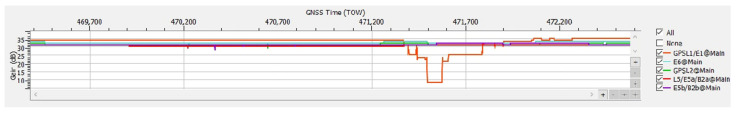
Test drive #4: **Upper half**—carrier-to-noise density C/N0 vs. time for Galileo E1 signals; **Lower half**—corresponding variation of the automatic gain control (AGC) value vs. time.

**Figure 10:**
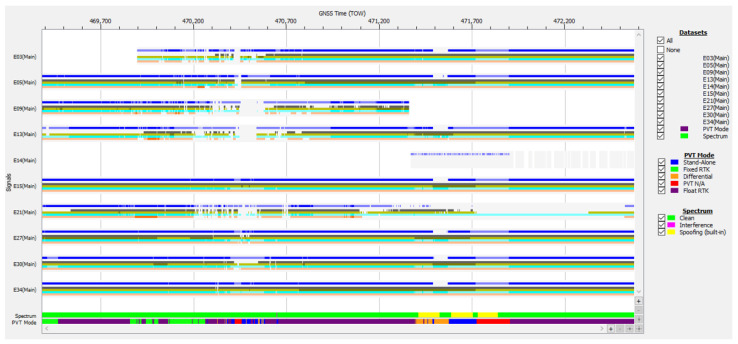
Signals of Galileo satellites tracked vs. time, test drive #4. Color legend for signal bands: blue—E1, dark green—E6, light green—E5a, cyan—E5b, pink—E5.

**Figure 11:**
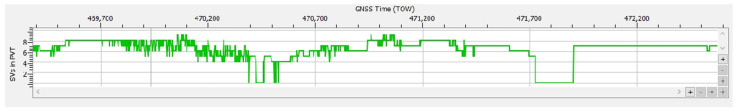
Number of authenticated Galileo satellites vs. time in PVT calculation.

The data logged during test drive #4 on 31 May 2024 are plotted in Figure 9, Figure 10 and Figure 11. Analyzing the plotted data revealed behavior specific to strong RF interference, as follows:

- The values of carrier-to-noise density ratio (C/N0) of the Galileo E1 signals received experienced a large drop for an approx. 470 s interval starting at TOW 471,373. C/N0 variation must be corroborated with the evolution of the automatic gain control (AGC) value of the front end of the receiver in the L1/E1 band. The main role of the automatic gain control (AGC) in the front end is to maintain the received signal at an optimal level for the analog-to-digital converter (ADC). This ensures that the signal is neither too weak, resulting in low signal-to-noise ratio, nor too strong, causing clipping and distortion;
- The value of the AGC dropped from about the usual 34 dB to approx. 8 dB in the same temporal interval. This indicated that the amount of signal energy coming into the receiver via its antenna was much higher than before, due to a large RF interference signal.
- It was observed that the value of AGC decreased only for the frequency L1/E1, and the gain remained stable for the other GNSS bands processed by the receiver, i.e., E6, L5/E5a, E5b. This indicated that the RF interference affected just the L1/E1 band;
- For the rest of the file, the AGC was stable at the nominal level of 34 dB, even if C/N0 also experienced brief drops. It can be safely concluded that these were due to situations involving low-elevation satellites, obstructions of the line of sight (LOS) to satellites, or multipath interference in the urban environment that the test car was driving through;
- The presence of strong RF interference in the E1 band was confirmed by the graph of signals from all nine Galileo SVs tracked, presented in Figure 10 in a deep blue color, displaying an 80 s interruption in tracking starting at TOW 471,484 The same graph confirms that signals in E5a, E5b and E6 were not affected;
- Another important indicator of the presence of RFI was revealed by the built-in interference and spoofing detection capability of the GNSS receiver used. The proprietary advanced interference monitoring and mitigation (AIM+) technology embedded by the designers can provide indications of interference, including spoofing, detection and mitigation [36]. The user is alerted in case of presumed spoofing by the setting of a special flag bit, based on a set of built-in tests to check the authenticity of the GNSS signals. In Figure 9, the spoofing alerts during the interval mentioned above are marked in yellow on the bar;
- Additional information is given in Figure 9 and Figure 10 with the presence of a signal corresponding to Galileo SV E14 that was received and tracked only for a period of approx. 521 s. This was a strong signal, approx. 52–52 dB-Hz, while all the other Galileo E1 signals experienced severe drops of more than 25 dB-Hz. This was definitely not a genuine signal transmitted by the E14 Galileo satellite, but probably a spoofed one.

As shown in Table 1, during the total of 3192 epochs of the recording on track #4, the receiver was able to report a strict-mode OSNMA-authenticated solution that included at least four authenticated Galileo satellites, for 92.98% of the time, under conditions of strong RFI that covered approx. 12% of the total time, demonstrating robustness and resiliency in conditions of severe interference.

Case study no. 2—RFI

**Figure 12:**
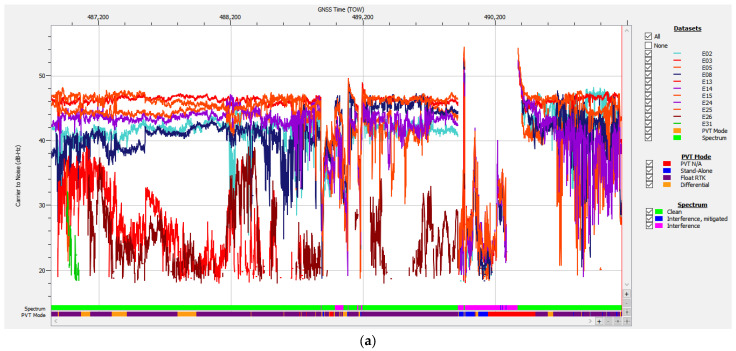

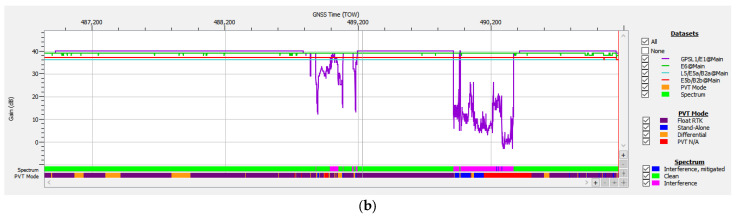
Test drive #15: (**a**) Carrier-to-noise density C/N0 vs. time for Galileo E1 signals; (**b**) corresponding variation in AGC value vs. time.

Taken from the data logged during test drive #15 on 13 September 2024, the graph of C/N0 and the AGC value of the Galileo E1 signals is presented in Figure 12.

The evolution of the carrier-to-noise density ratio of the Galileo E1 signals corroborated by the diagram of AGC values of the receiver in the same band show another typical case of high-power RFI, which occurred in two bursts that disturbed the tracking, ranging, and PVT calculations. However, in comparison with the signals in case study #1 and using the same methods of analysis, results indicated that there were no spoofed signals this time, as all E1 signals experienced similar drops in C/N0 values and there was no abnormal SV signal with C/N0 higher than the average.

**Figure 13:**
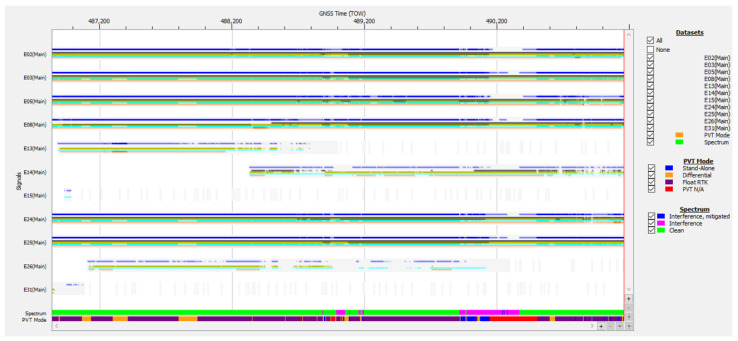
Signals of Galileo satellites tracked vs. time, test drive #15. Color legend for signal bands: blue—E1, dark green—E6, light green—E5a, cyan—E5b, pink—E5.

**Figure 14:**
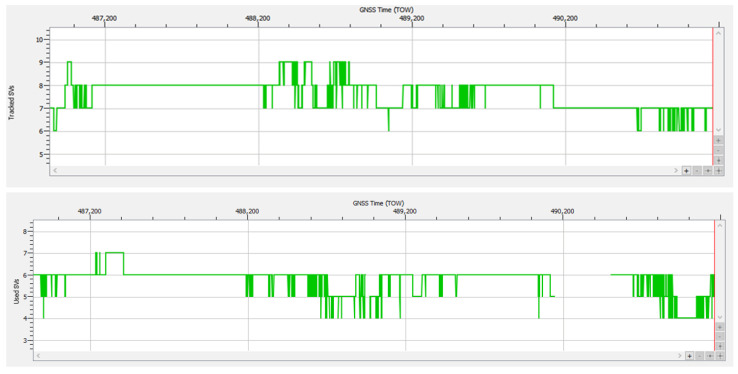
Test drive #15: **Upper half**—number of Galileo satellites in track vs. time during; **lower half**—number of Galileo satellites in PVT calculation vs. time.

The AGC plot in Figure 12 and the Galileo signals plot in Figure 13 show that both the first RFI burst, present for aprox. 330 s starting at TOW 488,840, and the second one, present for aprox. 460 s starting at TOW 489,915, focused only on the E1 band. The values corresponding to bands E5a, E5b, and E6 were constant and not affected by the disturbances. Combined analysis of Figure 12, Figure 13 and Figure 14 gives a clear picture of the impact of the two RFI bursts on the number of authenticated SVs and the subsequent loss of PVT. During both RFI events, the E1 signals received from satellites E02, E03, E05, E08, E24, and E25 were severely disturbed by the energy of the interfering signal, which led to PVT loss for 31 s starting at TOW 488,943 and for 357 s starting at TOW 490,145. Regarding the presence of the Galileo E14 SV signal in Figure 13, it should be noted that the Septentrio receiver did not include the E14 SV, which was declared by EUSPA as not usable, in any ranging or PVT calculation.

**Figure 15:**
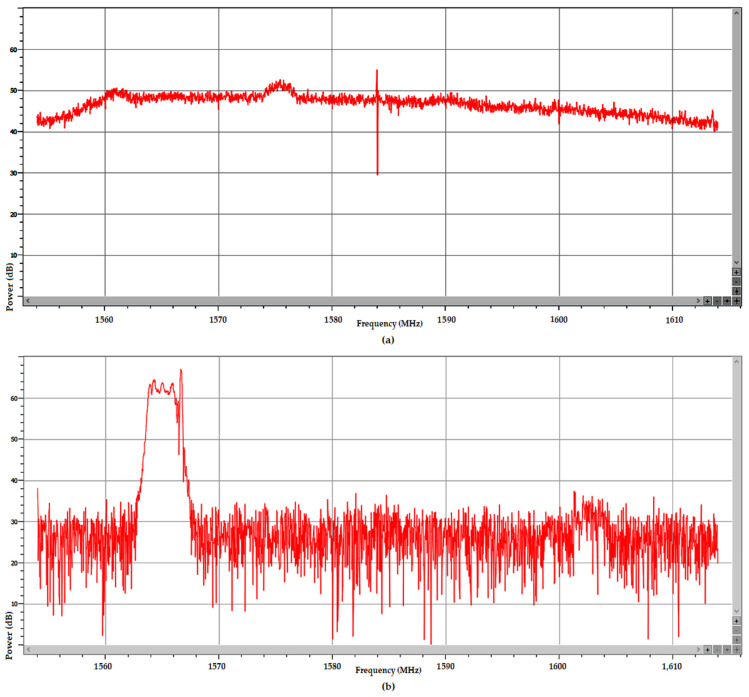
(**a**) Normal power spectrum of Galileo signals in E1 band, averaged over 15 scans (vertical line marks 1584 MHz); (**b**) Instantaneous power spectrum of RF interference signal, adjacent to E1 band recorded around TOW 490,200.

In the frequency domain, the normal power spectrum of the L1/E1 band as usually received during the test drive in absence of RFI is presented in Figure 15a, while the power spectrum of the RF interference signal at an instance in time around TOW 490,200 is presented in Figure 15b.

**Figure 16:**
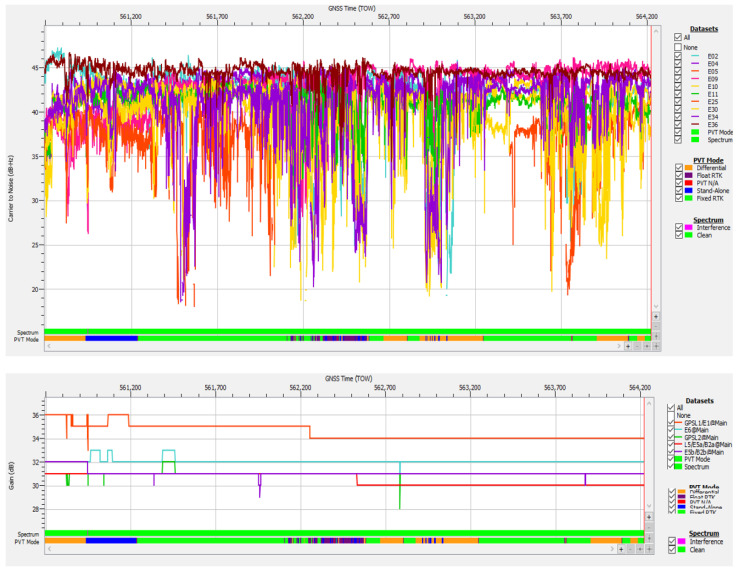
Test drive #13: **Upper half**—Carrier-to-noise density C/N0 vs. time for Galileo E1 signals; **lower half**—Corresponding variation of AGC vs. time.

Figure 15 is just a snapshot, but examination of the recorded signal power spectrum over the full length of the time interval when interference was active showed that the RFI signal swept an approx. 10 MHz bandwidth between 1560–1570 Mz. Due to the large power level of the RFI signal of almost 40 dB relative to noise, the E1 signal situated at only 5 MHz difference was still overpowered and the AGC in the front end of the receiver pushed down by 30–40 dB to compensate, as shown in Figure 16. The same figure proves that this interfering signal did not include significant components in the E5a, E5b, and E6 bands, as the AGC for these remained stable.

Despite the high level of RFI, the receiver maintained tracking of at least six Galileo satellites, with an average number of 7.61 satellites throughout the 4323 s of the test drive. This proves that the built-in interference mitigation capability of the receiver performed successfully. Nevertheless, the data necessary for OSNMA were corrupted for 391 s, so a PVT solution could not be calculated as signals from less than four satellites were authenticated. It should be noted that the strong RFI signal presence of RFI also led to errors or total loss of the pseudorange and phase measurements in the E1 band, which also resulted in loss of PVT.

Statistics for track #15 indicate the availability of an authenticated navigation message and a corresponding PVT solution for approx. 91% of the 4324 s of the whole recording, while strong RF interference was present for at least 12% of the total duration.

Case study no. 3—environment with hills and forested areas

The data in Table 1 indicate that not all files logged during test drives contained evidence of strong RFI that disrupted satellite signals and interfered with OSNMA processing. One such file is track #13, recorded on 1 June 2024 and analyzed below to assess the performance of OSNMA processing during a test drive in a complex environment unaffected by RFI.

As depicted in Figure 16, the C/N0 of the Galileo signals experienced significant variations of up to 25 dB when driving through the forested hilly areas or tree-lined roads that were the predominant environment of the road during the test drive. These features created challenges for signal reception due to LOS blockages, reflections (multipath), and attenuation.

**Figure 17:**
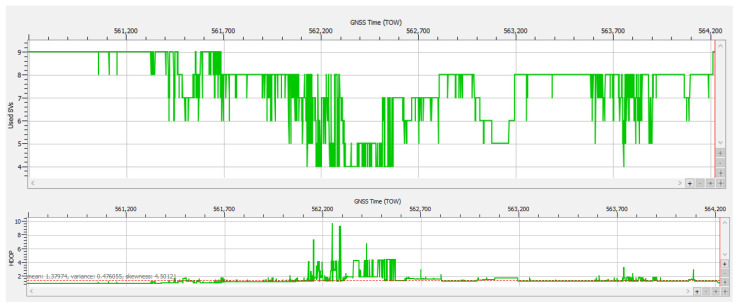
Test drive #13: **Upper half**—number of Galileo satellites in PVT vs. time; **lower half**—position’s dilution of precision vs. time.

The values of the AGC in Figure 16 present only a minor variation of ±3 dB for all received bands, which means that there was no sign of RFI and the receiver performed well in given conditions. Figure 17 shows that during the whole duration of the test drive, at least eight Galileo satellites were tracked, with nine being the maximum number attained.

The number of authenticated satellites was at least four in 99.86% of epochs, but with a good average of 7.51 satellites. Overall, for only 5 s during the recording’s whole duration of 3531 s was a PVT not available, representing very good performance in an environment with frequently poor satellite visibility and multipath conditions.

The variability in the number of authenticated Galileo satellites was influenced not only by potential RFI but also by satellite visibility, which was affected by the fact that the antenna was mounted on a moving vehicle, leading to variations with time and location. However, when the PVT solution used only four or five authenticated satellites, this minimal number of satellites resulted in poor dilution of precision (DOP), as can be observed in Figure 17, leading to bad positioning accuracy, as discussed in the next section.

## 5. Discussion

### 5.1. Presence of RFI

From the planning stage, the present research was focused on the impact of RFI on the operation of an OSNMA-enabled receiver relying exclusively on authenticated Galileo-based positioning. Our study did not aim to detect possible RFI in real time from the live received signal during the actual test drives, nor to conduct RFI classification, nor to identify the source of interference and triangulate its location. For these reasons, there was no specialized equipment in the test vehicle to accomplish such tasks. Identification and evaluation of interference events was carried out only in post-analysis using data collected by the GNSS receivers and logged as SBF files during the test drives, afterwards examined with dedicated software.

As previously mentioned in Section 4, this process of identification of interference incidents relied on two key metrics: C/N0 and AGC. Decrease of the carrier-to-noise density ratio (C/N0) of a received signal is a generally used indicator to assess the impact of interference, as a drop in C/N0 usually indicates its presence. However, relying solely on low C/N0 values is insufficient, since factors such as signal obstructions, multipath effects, or receiving signals from low-elevation satellites can also lower C/N0 [37].

The AGC measurement indicates the level of incoming power to the receiver [38,39,40]. When RFI occurs, whether due to jamming or spoofing signals, the thermal noise floor rises, causing the AGC to reduce its gain, resulting in a lower value. C/N0 typically decreases in the presence of interference; however, during a spoofing attack, the falsified signal is designed to exceed the value of the authentic GNSS signal, which might cause the C/N0 to maintain the same value or even increase. Therefore, on one hand, if both AGC and C/N0 decrease, jamming is suggested, as in Figure 12 and Figure 13. On the other hand, if the AGC drops but C/N0 increases significantly for only one signal, this could be an effect of spoofing, as was the case for the SV E14 signal in Figure 9 and Figure 10.

Detailed analysis of the logged data, focusing on the modifications in C/N0 and AGC values, revealed many details in several cases of RFI that were encountered. Out of the 18 data files logged, each corresponding to a segment of road driven by the test vehicle, 10 files presented indications of interference affecting between 2% and 29% of the duration of recording. The duration of each RFI incident was estimated on the C/N0 and AGC graphs, with a certain margin of error. Overall, the RFI affected approx. 5.28% of the total 23.9 h of recorded data.

The equipment set-up used did not include a separate digital RF GNSS signal recorder/player. However, the data block labeled BBSamples, contained in the SBF file logged during the test drive, stored successive baseband samples taken at the output of the receiver’s analog-to-digital converters. Using the Septentrio software RXControl v23.1 [35], these samples allowed playback and display of the recorded signal power spectra, separately for each of E1, E5, and E6 bands.

**Figure 18:**
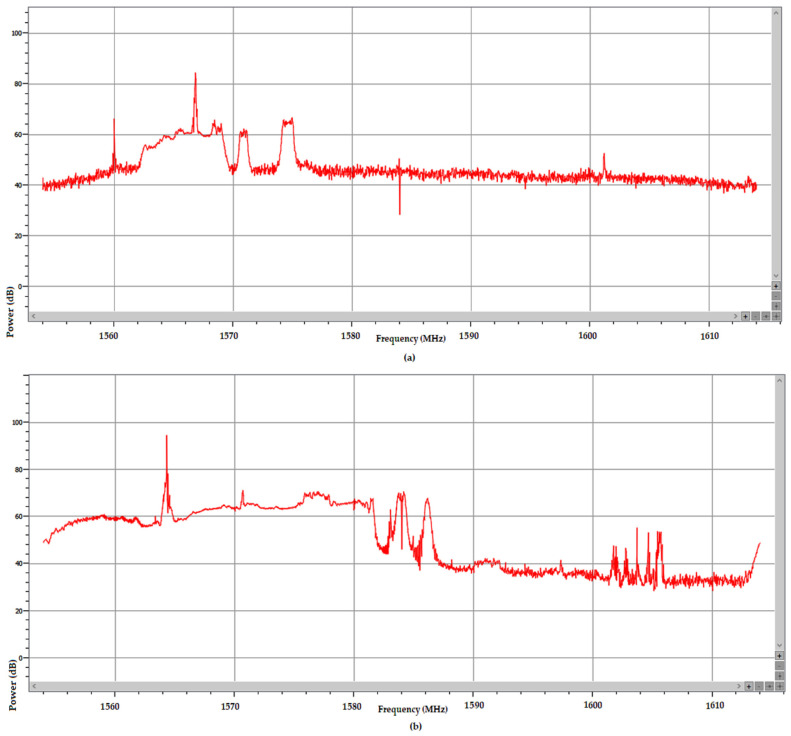
Power spectra of RFI events recorded during test drive #14. Center frequency of plots is 1584 MHz. (**a**) Wide-band RFI affecting GPS L1 and Galileo E1 band; (**b**) Wide-band RFI affecting L1/E1 and Glonass G1 bands.

Analysis of these spectrum display plots gives indications about the type and possible source of RFI events that were experienced during the test drives and recorded in the SBF files. Figure 18 displays the power spectra versus frequency for two recorded RFI events during test drive #14 on 13 September 2024. The power spectra were averaged over 20 sweeps and show the full bandwidth of the interfering signals, which had different characteristics in each of the events, probably corresponding to different sources of interference. The interference in the upper half of Figure 18 covered the Beidou band centered on 1561 MHz and the L1/E1 band centered on 1575 MHz, while the RFI in the lower half of Figure 18 has a wider bandwidth and also disturbed the Glonass G1 band 1589–1605.37 MHz.

Analysis of the same spectrum samples in the Galileo E5 and E6 frequency bands demonstrated that these were not affected by RF interference.

Regarding the possible sources of RFI signals such as were recorded during the test drives, it was observed that the profiles of the power spectra in Figure 18 were similar to those already presented and analyzed in studies regarding the detection, monitoring, classification, and mitigation of GNSS interference [41,42,43]. These reports indicate that the usual sources of such interference are so-called personal privacy devices (PPDs), which are cheap and already widespread, providing means to disrupt the reception of GNSS signals in a localized area in the vicinity of the user, with the purpose of preventing their location from being tracked, to counter technical surveillance or impede location data collection by apps in mobile devices, etc. In our data-collection campaign, strong RFI events were encountered when the test vehicle was driving or parked close to heavy lorries or in the vicinity of river vessels, so it is therefore likely that such devices or other types of RF signal sources were being used by those parties.

### 5.2. Effects of RFI on Processing of OSNMA Data

The impact of RFI incidents such as those described in Section "Data Content of Logged SBF Files" and Section 5.1 on the calculation of PVT solution is twofold. First, when the C/N0 is low enough, the receiver cannot acquire and track the SV signal any more and does not generate ranging measurements; thus, the position solution cannot be computed using the E1 signal. Secondly, the strong interfering signal in the E1 band causes the receiver to stop correctly demodulating the I/NAV data and drop pages and full sub-frames of navigation messaging, leaving insufficient data to perform correct decoding of the OSNMA information. The overall authentication algorithm performance is reduced and the number of authenticated satellites decreases below the minimum of four for a PVT solution, leading to full loss of strict-mode OSNMA-authenticated navigation solutions.

The performance of OSNMA decoding in the presence of strong RFI is also influenced by the strategy implemented by the receiver to retrieve and process the I/NAV data necessary for authentication (DSM, TESLA chain keys, tags, and navigation data), focusing on optimizing data collection in varying signal conditions [16]. Such strategies could be sub-frame based, page-based, sequential from one satellite, or parallel over multiple satellites in view. For efficient operation during RFI events, it is best that the OSNMA implementation extracts any usable data from the sub-frame, incomplete or not. From this point of view, it is better to process the data on a page level, instead of a sub-frame level. While the exact methods applied by the Septentrio Mosaic receiver for OSNMA processing are not disclosed to the user, the resulting PVT solutions during strong RFI in case studies #1 and #2 are examples of its robustness and also of its limits.

Operation in both the E1 and E5 bands allowed the receiver to output a valid PVT solution for part of the duration of the strong RFI, as the measurements were obtained using processing in the E5 band while the authentication data for at least four satellites were still valid. In the event plotted in Figure 9, as the RFI signal became stronger starting at approx. TOW 471,450, the number of authenticated satellites included in the PVT solution started to drop from an average of 7 to 6, then 5, then less than 4, and the PVT solution was lost completely. After this moment, for position/time solutions, the receiver was not able to rely on the good signals that remained in the E5 and E6 bands, because it was operating in OSNMA strict mode that required processing of E1 signals only from authenticated SVs.

### 5.3. Assesment of Key Performance Indicators

The assessment of the overall performance of the OSNMA-enabled receiver was carried out using the key performance indicators (KPIs) described in the following list:

OSNMA PVT availability

One of the KPIs for the user of an OSNMA-enabled GNSS receiver operating in strict mode is the availability of a PVT solution. This indicator represents the percentage of the full length of the dataset during which a PVT solution can be computed using only Galileo data-authenticated satellites with a healthy SIS [13]. According to Table 1, the availability of an OSNMA-authenticated PVT solution, calculated at 1 Hz rate for all epochs during the data-collection campaign, was 95.18%. This demonstrated the very good performance of the OSNMA-enabled receiver and of the algorithm itself, considering the fact that the strong RF interference that disrupted the decoding of I/NAV data blocks was present for approx. 5.28% of the total time. As well as RFI, signal obstructions, multipath conditions, and the poor environment contributed to additional degrading of satellite availability, but the OSNMA data decoding and processing demonstrated robustness, and authenticated PVT was lost to a lesser extent due to these factors.

**Figure 19:**
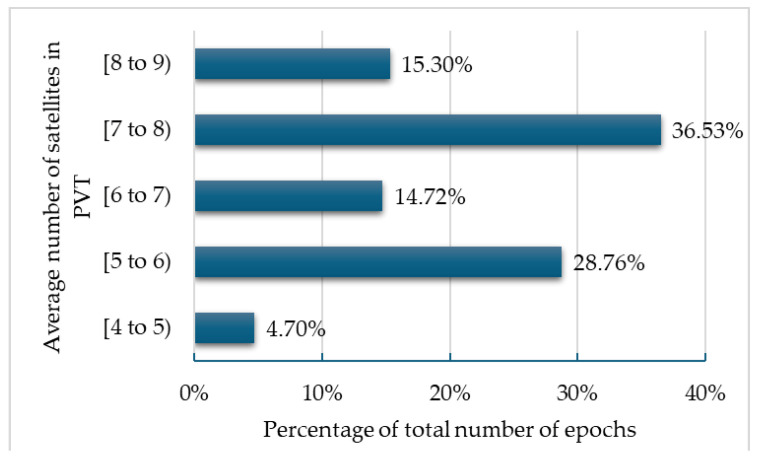
Distribution of average numbers of successfully authenticated satellites for all 86,046 epochs.

PVT availability was close to the percentage of availability of at least four authenticated satellites, which was 95.42% of all epochs. The average number of authenticated satellites included in the PVT for all epochs was 6.7. The breakdown of this value for average numbers between four and nine satellites available is shown in Figure 19, where the notation [4 to 5) means four to five satellites.

As expected, the best PVT availability results of 100% were obtained in open-sky rural terrain and at time intervals when the number of available Galileo satellites was about highest possible for the geographical coordinates of the location. For example, in test drive #11, on 1 June 2024, for 3256 epochs, the Galileo almanac indicates visibility of 10 to 11 SVs, the logged SBF file reported that the receiver tracked an average number of 9.67 Galileo satellites, and the OSNMA processing authenticated an average number of 8.07 satellites. Despite the presence of RFI for approx. 1.90% of the total duration, authenticated PVT was provided for a full 100%, demonstrating the robustness of the OSNMA data retrieval and algorithm processing (Table 1).

A worst-case scenario of degraded PVT availability resulted from the analysis of test drive #8, on 31 May 2024, for 4042 epochs (Table 1), when the road crossed an environment that included only about 50% open-sky terrain, the rest being tree-lined rural, peri-urban, or urban, with no RFI events being recorded. According to the almanac, there were between five and seven visible satellites, but due to presence of LOS signal obstructions, attenuation, and multipath conditions, an average of only five were tracked by the receiver and an average of 4.29 were authenticated and used in PVT. These were the lowest average numbers of authenticated satellites obtained throughout the data-collection campaign but still larger than the minimum required of four. The obstructions led to a drop in PVT availability to 86.89% of the test drive’s total duration, even though there was no significant RF interference. This demonstrated that a reduced number of available Galileo satellites did not allow for redundancy in the received and processed OSNMA data messages, leading to degradation of more than 10% in terms of availability under adverse environmental conditions, in comparison to open sky.

OSNMA PVT accuracy

This KPI refers to the accuracy of the position solutions calculated from measurements in strict mode using only Galileo data-authenticated satellites with healthy SIS. OSNMA was designed to ensure that its implementation would have a minimal effect on the accuracy and performance of the PVT solution, compared with the standard open service [44]. Similar published studies on the experimental assessment of OSNMA performance [17,18,19] have relied on inertial measurement unit (IMUs) to provide the ground-truth reference values necessary for calculating the accuracy. The present study used just a commercial off-the-shelf (COTS) GNSS receiver, without IMU, to provide a posteriori accuracy estimates of computed position based on the assumed measurement noise model and values of C/N0. The accuracy values reported by the Mosaic-X5 receiver were theoretical predictions based on internal modeling, not direct measurements of the actual position error. While these estimates are useful for understanding the quality of the computed position under assumed conditions, they may not always align perfectly with real-world performance, due to external influences, particularly under multipath conditions.

Throughout our data-collection campaign, the GNSS receiver was configured to apply RTK correction messages, producing either RTK-fixed or RTK-float positioning estimates according to whether it could resolve the carrier phase integer ambiguities. This setup yielded accuracy to within several centimeters in fixed mode and several decimeters in float mode [28]. If RTK solutions were unavailable, the receivers were set to default to differential or stand-alone positioning modes.

As a reference for relative a posteriori calculated accuracy for performance comparison, we use the results of an additional data-collection campaign that we also carried out for several days with the same GNSS receiver and antenna in a static position in Bucharest, under optimal clear-sky conditions, between 8–11 September 2024. The receiver was set to operate in OSNMA strict mode (Galileo only) and received an uninterrupted stream of RTK messages in RTCM format, enabling PVT computation in RTK-fixed mode. The statistics for the a posteriori calculated accuracy for a logged file of 20,904 epochs at 1 Hz, i.e., 5.8 h, are presented in Table 2 and can be considered as a best-case example of accuracy obtained in OSNMA strict mode, RTK-fixed mode.

During the data-collection campaign with the test vehicle, internet access was sometimes inconsistent, leading to periods when the GNSS receiver was unable to obtain the RTCM correction messages needed for RTK positioning, especially on 30 May–1 June when connection to the internet was provided via a mobile phone in the test vehicle. For this reason, due to the non-homogeneous conditions under which the tests were carried out, it would not be correct to make statistical averages of the accuracy of the entire dataset. Instead, we next analyze the accuracy results for two individual test drives presented in Table 1.

**Figure 20:**
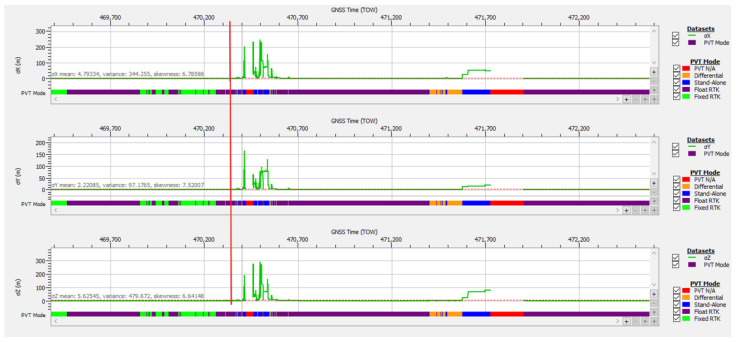
Positional accuracy: standard deviation time plot for test drive #4, case study #1. The red vertical line marks the first 840 s of the recording.

For the signal logged during test drive #4 and analyzed as case study #1 (spoofing), which included 224 s of PVT loss due to signal obstructions, multipath, and RFI disturbance, the estimate for a posteriori calculated accuracy is displayed in Figure 20. Examination of the graphs shows that, with the exception of two signal degradation events at TOW 470,380 and TOW 471,572, the accuracy values lie within the usual range for average signal quality conditions, i.e., 2 to 5 cm for RTK-fixed and 0.2 to 0.6 m for RTK-float mode, on each of the three axes. The statistics for the full duration of the logged file in test drive #4, shown in Table 3, are degraded by the expected poor values that resulted during the two events of strong RFI, LOS obstacles, and multipath conditions.

**Figure 21:**
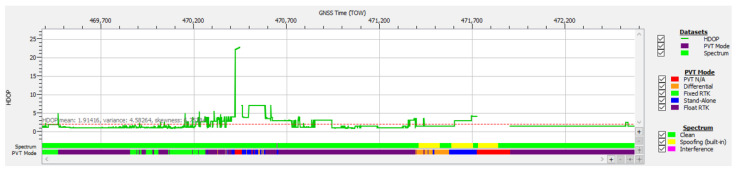
Horizontal dilution of precision time plot for test drive #4, case study #1 (spoofing).

The degradation of positional accuracy over some intervals is explained by the presence of only four or five authenticated satellites included in the PVT, as shown in Figure 11, resulting in high values for the horizontal dilution of precision (HDOP) as plotted in Figure 21.

To obtain a more realistic image of the positioning performance in OSNMA strict mode in areas without severe signal degradation, a segment of file should be analyzed: for example, the first 840 s of the file till the red vertical line marked in Figure 20. The statistics for the reduced file dataset are presented in Table 4 and show clearly improved performance, comparable to the results obtained under optimal conditions listed in Table 2. For this reduced dataset, the average number of satellites included in the PVT was 7.2, resulting in an average HDOP of 1.2 and good accuracy performance.

**Figure 22:**
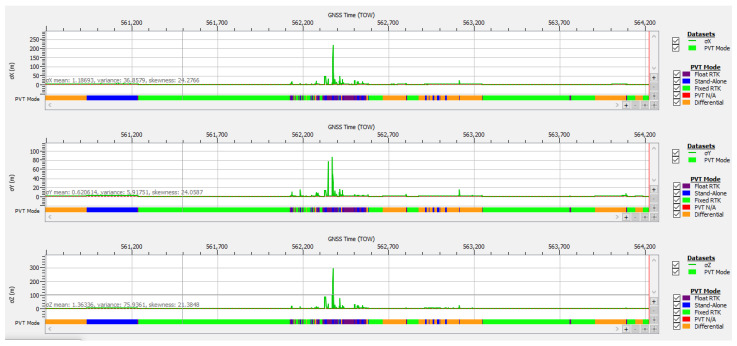
Positional accuracy: standard deviation time plot for test drive #13, case study #3 (obstructions due to environment).

In case study #3 (obstructions due to environment) also presented in the previous Section “Data Content of Logged SBF Files”, the signal logged during test drive #13 was not affected by RF interference but only by poor satellite visibility, attenuation, and multipath conditions that resulted in a 5 s loss of PVT around TOW 562,380. The plot of the estimated accuracy‘s standard deviation shown in Figure 22 and the statistical values presented in Table 5 confirm that the positioning performance was much better than in case study #1. For approx. 97.2% of the duration of test drive #13, the receiver was able to provide the nominal accuracy specific to all PVT modes, stand alone, differential, float RTK and fixed RTK, in accordance with the design goal of OSNMA.

The degradation of accuracy in the approx. 100 s interval centered on TOW 562,386 was not due to RFI, as in case study #1 (spoofing), but to the increased DOP in the same interval, because of a low number of satellites in PVT, only four or five, as shown in Figure 17. This confirms the importance of a high number of Galileo satellites available not only for PVT availability, as discussed earlier, but also for maintaining good accuracy for positioning even under difficult environmental conditions.

### 5.4. Future Developments in Signal Authentication and Strengthening GNSS Signals Against Interference

The analysis carried out in the previous sub-sections demonstrates that, at present, the main option to improve performance of the two KPI is to increase the numbers of available Galileo satellites in the constellation and of those that broadcast OSNMA data. These possibilities are certainly limited by the design of the Galileo system; one could reasonably expect just a slight increase of one more satellite in addition to the already good average numbers of 8.35 tracked and 6.7 authenticated Galileo satellites that were obtained in our research.

A radical improvement could be accomplished by the introduction of cross-authentication between Galileo and GPS satellite signals. Consequently, the number of authenticated satellites available to an OSNMA-enabled receiver would potentially double and the calculation of the PVT would benefit from more options for optimal satellite configurations with improved DOP, resulting in much improved accuracy under conditions where reception is currently challenging. Cross-authentication between GNSS constellations like Galileo and GPS has been discussed as a potential future enhancement for interoperability, but such capabilities would require significant technical and political collaboration.

Galileo receivers can enhance anti-spoofing security by integrating OSNMA with methods such as monitoring signal power, evaluating signal quality and consistency, or employing hardened future signals featuring spreading code authentication. Examples include the upcoming encrypted Galileo E6-C component, specifically designed for signal authentication, and the GPS Chips-Message Robust Authentication (CHIMERA) system. CHIMERA is an enhancement to the GPS L1C signal designed to authenticate navigation data and provide encryption for the spreading codes. Currently, CHIMERA is in the testing phase [45]. Additionally, positioning, navigation, and timing (PNT) applications can incorporate alternative radionavigation or timing systems as backups to improve reliability and resilience against attacks.

Other key areas of improving the OSNMA protocol and strengthening GNSS signals against interference need to be addressed. For example, one of the main challenges with OSNMA is the delay in obtaining the first authenticated position fix. Optimizing the time to first authenticated fix (TTFAF) can significantly enhance the user experience. This can be achieved by processing partial information from broken sub-frames and intelligently using fields in the authentication tags to reconstruct missing navigation data. Also, the TESLA protocol used in OSNMA can be further optimized for better performance and security, for example, by exploring new cryptographic algorithms and improving the efficiency of key management and distribution.

## 6. Conclusions

We carried out tests on various roads, in open-sky, rural, and urban settings, and under RF interference, in counties in east and south-east Romania, in order to evaluate the performance of GNSS positioning using only OSNMA-authenticated Galileo signals. Considering the results obtained from capturing and analyzing Galileo OSNMA signals and positioning solutions, this study extends and complements similar reports [17,18,19,46], but to the best of the authors’ knowledge, it is the only research that has considered strong RF interference monitored at close range in real-life scenarios.

The presence of strong RFI incidents, most probably intentional, is in accordance with initial research on the area chosen for test drives and confirms that the Black Sea area is increasingly a risk area for the use of GNSS-based navigation, positioning, and timing. Actually, additional studies published since the inception of the present research substantiate this conclusion, as more jamming and spoofing incidents have been reported [47,48,49].

During the data-collection campaign, which lasted approximately 23.9 h, the receiver was able to provide a PVT solution using only authenticated satellites for 95.18% of this time, while RFI affected approx. 5.28% of the total duration. This level of availability is quite high for the terrain conditions of the roads travelled and the challenging RF environment and should be attributed to two main aspects: first, the high level of performance of the interference detection and mitigation technology employed by the GNSS receiver; and second, the robustness of operation of the OSNMA algorithm, which was still in the observation phase. The strong RFI encountered during the test drives disrupted only the E1 band component of the Galileo signals, and the triple-frequency receiver used was able to maintain tracking and provide measurements for PVT during such events using the E5 band. However, when authenticating at least four satellites, it was suppressed due to degraded I/NAV data, and the solution was dropped completely. This proves that increasing the survival of OSNMA processing during RFI events requires more Galileo satellites transmitting authentication data. The average number of simultaneous authenticated satellites was 6.7, which was approx. two satellites fewer than reported in similar studies carried out in optimal open-sky conditions and without any reported RF interference. Judging from the gradual process of degrading the OSNMA decoding during RFI, it is expected that an additional number of authenticated SVs acquired before beginning of the disturbance event would maintain a valid PVT for longer time and increase resiliency and the availability of KPIs.

The study also shows that use of only OSNMA-authenticated SVs in conjunction with reliable connection to RTCM provides very good and stable PVT availability and positioning accuracy in RTK modes, as long as the number of available authenticated satellites reaches at least seven or eight. Such progress may be anticipated as additional Galileo satellites are launched and the number of those transmitting OSNMA data consequently increases. A real breakthrough in improvement of authenticated GNSS navigation would be the planned Galileo–GPS cross-authentication allowed by the OSNMA protocol. When or if cross-authentication capabilities are fully developed and supported by both systems, dual-constellation receivers would benefit from an increased number of authenticated satellites, potentially improving positioning availability, accuracy, and robustness, especially in challenging environments.

The logged signal files and the performance of the OSNMA algorithm were analyzed using only the COTS software v23.1 provided by the manufacturer of the GNSS receiver, which did not include detailed results of the intermediate stages of processing and decoding of data blocks—data words, keys, MACs, and tags—or about the decision logic for establishing the authentication status for a certain satellite. Using dedicated open-source tools such as OSNMAlib [13,14] or FGI-OSNMA [26,46] to process the collected dataset will certainly provide a better understanding of how the authentication mechanism is degraded by RF interference and, equally importantly, how long it takes the receiver to recover and again determine the authentication status after a PVT interruption due to RFI disturbance. In a follow-up to the present paper, using the abovementioned open-source software, the authors intend further to analyze the dataset obtained in the present research, including in OSNMA loose mode, enhanced with a new data-collection campaign and taking advantage of the planned declaration of full operational development of the Galileo OSNMA service.

## Figures and Tables

**Table 1:** Values of interest for each SBF file logged during test drives and averages over total duration of data-collection campaign.

1	2	3	4	5	6	7	8	9
Test Drive/Track #	Date	Duration (s) /Number of 1 s Epochs	Length of Track (km)	Duration of RFI (% of Test Drive Duration)	PVT Available (% of Test Drive Duration)	No PVT Available (% of Test Drive Duration)	Mean Number of Galileo SVs Tracked	Mean Number of Galileo Authenticated SVs in PVT
1	30 May 2024	4141	61	0.5	99.90	0.10	6.81	6.61
2	30 May 2024	2026	16	0	100.00	0.00	6.19	6.05
3	31 May 2024	3306	21	0	100.00	0.00	7.37	6.22
4	31 May 2024	3192	16	12	92.98	6.24	8.44	6.26
5	31 May 2024	3228	2	2.32	92.24	2.76	8.28	7.57
6	31 May 2024	3368	56	13.39	99.97	0.03	7.79	7.22
7	31 May 2024	3944	13	29.00	68.23	31.77	6.16	5.65
8	31 May 2024	4042	28	12.00	86.89	13.11	5.00	4.29
9	31 May 2024	1107	0	0.00	100.00	0.00	5.51	5.13
10	31 May 2024	3736	24	13.22	96.44	3.56	7.30	5.44
11	1 June 2024	3256	7	1.90	100.00	0.00	9.67	8.07
12	1 June 2024	740	12	0.00	99.86	0.14	8.00	7.51
13	1 June 2024	3531	43	0.00	99.86	0.14	8.61	7.52
14	13 September 2024	9906	104	7.65	92.94	7.06	9.85	8.06
15	13 September 2024	4323	12	12.02	90.96	9.04	7.61	5.74
16	13 September 2024	2520	35	0.10	99.92	0.08	8.65	5.86
17	14 September 2024	20,567	156	3.08	98.82	1.18	9.57	7.37
18	14 September 2024	9113	149	0.00	95.53	4.47	8.60	5.76
**Totals and average values**		**86,046**	**755**	**5.28**	**95.18**	**4.82**	**8.35**	**6.7**

**Table 2:** Summary statistics of the a posteriori calculated accuracy values with static receiver position under clear-sky conditions, 20,904 epochs, OSNMA strict mode, RTK-fixed mode.

Data	Minimum (m)	Maximum (m)	Mean (m)	Std. Dev (m)
Σx	0.0128	0.0252	0.0180	0.0026
Σy	0.0123	0.0309	0.0157	0.0030
Σz	0.0157	0.0335	0.0196	0.0032

**Table 3:** Summary statistics of the a posteriori calculated accuracy values of positions in case study #1 (spoofing), full length of logged file 2967 epochs, including signal degradations due to RFI, LOS obstacles, and multipath conditions.

Data	Minimum (m)	Maximum (m)	Mean (m)	Std. Dev (m)
σx	0.0271	242.997	4.793	18.554
σy	0.0193	163.193	2.221	9.857
σz	0.0295	288.798	5.625	21.901

**Table 4:** Summary statistics of the a posteriori calculated accuracy values of positions for reduced-length data file of 840 epochs in case study #1, without signal degradations and with no RF interference.

Data	Minimum (m)	Maximum (m)	Mean (m)	Std. Dev (m)
σx	0.0271	0.1498	0.0424	0.0138
σy	0.0193	0.2482	0.0409	0.0244
σz	0.0295	0.1369	0.0478	0.0154

**Table 5:** Summary statistics of the accuracy values in case study #3 (obstructions due to environment), full length of logged file 3526 epochs at 1 Hz, including signal degradations due to attenuation, LOS obstacles, and multipath conditions.

Data	Minimum (m)	Maximum (m)	Mean (m)	Std. Dev (m)
σx	0.0312	215.872	1.1869	6.0710
σy	0.0185	85.5953	0.6206	2.4325
σz	0.0248	288.003	1.3633	8.7141

## Data Availability

Selected samples of the data collected will be made available to interested parties by email request to the corresponding author.
